# Linguistic law-like compression strategies emerge to maximize coding efficiency in marmoset vocal communication

**DOI:** 10.1098/rspb.2023.1503

**Published:** 2023-09-27

**Authors:** Cristina Risueno-Segovia, Deniz Dohmen, Yasemin B. Gultekin, Thomas Pomberger, Steffen R. Hage

**Affiliations:** ^1^ Neurobiology of Social Communication, Department of Otolaryngology—Head and Neck Surgery, Hearing Research Centre, University of Tübingen, Medical Center, Elfriede-Aulhorn-Strasse 5, 72076 Tübingen, Germany; ^2^ Werner Reichardt Centre for Integrative Neuroscience, University of Tübingen, Otfried-Müller-Str. 25, 72076 Tübingen, Germany; ^3^ Graduate School of Neural & Behavioural Sciences - International Max Planck Research School, University of Tübingen, Österberg-Str. 3, 72074 Tübingen, Germany

**Keywords:** communication, vocalization, linguistic laws, Zipf′s law, vocal compression, human language evolution

## Abstract

Human language follows statistical regularities or linguistic laws. For instance, Zipf's law of brevity states that the more frequently a word is used, the shorter it tends to be. All human languages adhere to this word structure. However, it is unclear whether Zipf's law emerged de novo in humans or whether it also exists in the non-linguistic vocal systems of our primate ancestors. Using a vocal conditioning paradigm, we examined the capacity of marmoset monkeys to efficiently encode vocalizations. We observed that marmosets adopted vocal compression strategies at three levels: (i) increasing call rate, (ii) decreasing call duration and (iii) increasing the proportion of short calls. Our results demonstrate that marmosets, when able to freely choose what to vocalize, exhibit vocal statistical regularities consistent with Zipf's law of brevity that go beyond their context-specific natural vocal behaviour. This suggests that linguistic laws emerged in non-linguistic vocal systems in the primate lineage.

## 1. Introduction

Human societies have been under evolutionary pressure to develop complex communication systems [1]. Language, as the apex of human social communication systems, emerged as a system of words and symbols used to exchange knowledge, share emotions and beliefs, and convey information about past and future events [2]. Under the need to transfer messages quickly and effectively using the available energy resources, signal compression arose. In fact, the organization of words follows statistical patterns defined as linguistic laws [3–5]. Zipf's law of brevity, for example, states that the more frequently a word is used, the shorter it tends to be [6]. This inverse relationship between frequency of use and signal duration is explained by the principle of least effort, a rule of coding efficiency that all human languages follow [3,7,8]. Yet, it is unclear whether such fundamental linguistic laws emerged de novo in human language or evolved from non-linguistic precursors [7–9]. While some studies find evidence for the law of brevity in primate vocalizations [10–12], others do not [13–15]. The main reason for this discrepancy might be that call types, exhibiting distinct call durations, are context specific and primarily encode internal states of arousal in primates [16,17]. Therefore, their relative occurrence, and thus call duration, is dependent on the corresponding behavioural context and is not necessarily subject to any statistical regularities that the pressure to produce a large number of vocal signals might induce.

The marmoset monkey, a small New World primate, has recently garnered considerable interest as a suitable model organism for studying evolutionary aspects of vocal motor control mechanisms underlying human speech [18,19]. In the last few years, several studies have observed different degrees of vocal flexibility allowing marmosets to cognitively control when to vocalize [20,21] and where to vocalize [22]. Furthermore, they have been shown to be capable of adaptively modifying distinct call features, such as call frequency, amplitude and duration, in response to external acoustical stimuli [23–25]. Recently, it has been revealed that marmosets exhibit synchronized and phase-locked phono-articulatory movements suggesting that such speech-like bi-motor oscillations, which are crucial for speech production, evolved early in the primate lineage [26]. While we cannot go back in time to observe evolution, we can recreate scenarios with selective pressures to find out how vocal systems adapt [1]. Here, we define selective pressure as the force that causes a particular vocal behaviour (in the present case, increasing call rate [27], decreasing call duration and increasing the proportion of short calls) to be more favourable under conditions of vocal reinforcement when animals have the freedom to choose whether to vocalize and what to vocalize. Thus, we experimentally mimicked conditions that monkeys would face under the selective pressure of increasing vocal signalling by rewarding every vocal utterance regardless of its type. We considered that if monkeys have the capacity to exhibit vocal compression patterns, evidence for brevity should emerge and develop when they are encouraged to increase the number of vocalizations in a goal-directed manner. We hypothesized that the cost and benefits associated with pre-determined call features may vary according to levels of volitional control. Higher vocal control might allow the detachment of distinct vocalizations from their original triggers and give monkeys the ability to choose what to vocalize independently of the invariant environmental set-up [28]. Therefore, we argue that during vocal training, monkeys will change their vocal behaviour towards a more rapid and concise vocal signalling to economize time and energy. We expect to observe a transition from long context-specific calls [29] to a more efficient coding of shorter calls as training progresses, despite the increase in task complexity. To test for Zipf's law of brevity, we consider three cases: (i) the tendency to articulate more calls in less time, (ii) the tendency of more frequent calls to become shorter or to be composed of fewer vocal motor units [30] and (iii) the tendency of shorter calls to be produced more frequently.

## 2. Methods

### (a) Experimental animals

We trained four adult common marmoset monkeys, *Callithrix jacchus*, two males and two females, in a vocal conditioning paradigm. Animals were born in captivity, housed at the University of Tübingen, Germany, and usually kept in different sex pairs. The facility room was maintained at approximately 26°C, 40–60% relative humidity and a 12 h : 12 h light/dark cycle. They had ad libitum access to water and were fed daily with standard commercial chow, fresh fruit and vegetables, mealworms and locusts. Marshmallows and special fruit (e.g. banana and grapes) were used to transfer the animals from their home cages to a transfer box. Experimental procedures were approved by the local authorities (Regierungspräsidium Tübingen; licence no.: CIN 01/20 G) and were in agreement with the guidelines of the European Community for the care of laboratory animals.

### (b) Data acquisition

Stimulus presentation, behavioural monitoring and reward presentation were synchronized and performed automatically using a custom-written program (OpenEX and Synapse, Tucker-Davis Technologies, USA) running on a workstation (WS-8 in combination with an RZ2 bioamp processor and RZ6D multi I/O processor, Tucker-Davis Technologies, USA) and a custom-written MATLAB program running on another PC, which was connected via an A/D interface card (PCIe 6321, National Instruments) with the workstation. A monitor screen connected to the desktop PC was positioned in front of the animal's head at a distance of 40 cm for visual stimulus presentation. Vocalizations were recorded using a microphone (MKH 8020 microphone with an MZX 8000 preamplifier, Sennheiser, Germany, in combination with a phantom power, PAN 48.2, Palmer) positioned 10 cm in front of the monkey's head and connected to a multi I/O processor (RZ6D, Tucker-Davis Technologies, USA). Vocalizations were recorded using the same system at a sampling rate of 100 kHz. Vocal onset and offset times were detected offline based on the spectrogram of the calls using software (Avisoft-SASLab Pro 5.2.13, Avisoft Bioacoustics) to ensure precise timing for data analysis. The spectrogram parameters used for our analysis were set to an FFT length of 1024 with a 50% overlap resulting in a temporal resolution of 52 429 ms. The monkey's behaviour was constantly monitored using a USB video camera (Brio, Logitech) placed in front of the monkey.

### (c) Behavioural protocol

We trained four marmoset monkeys (two males, two females; mean age 21.3 ± 2.9 months at start of training) in a vocal conditioning paradigm where they sat in front of a monitor in a soundproof chamber and were required to vocalize after cueing by an arbitrary visual stimulus [20]. The animals were trained with positive reinforcement to be voluntarily transferred from their home cage to the experimental room. Additionally, they were habituated to the experimental room by positive reinforcement before starting the vocal training. To achieve the performance of such complex behavioural tasks, monkeys were trained in discrete steps with a progressive increase in cognitive demand until they reached the final task. As previously described, training took place in a soundproof chamber where the monkeys were seated in a primate chair on their hind legs, with their tail in a natural and relaxed position. Vocalizations were recorded in three training stages (electronic supplementary material, figure S1*a*). In stage 1, animals were able to calmly sit in the primate chair and were rewarded for every vocal utterance. In stage 2, animals self-initiated trials by pulling a lever. They were rewarded when vocalizing upon presentation of a red squared visual cue with a duration of 10 s. This vocalizing window got gradually reduced to 3 s through stage 2. Stage 3 consisted of a visual go/no-go detection protocol. In addition to the stage 2 requirements, in stage 3 animals were rewarded when they successfully withheld their vocal output during the precue and only vocalized during the presentation of the go-stimulus. Importantly, the red cue (go-signal) only limited the time to initiate a vocalization and had no effect on call duration. If the vocalization started during the go-signal, the presentation of the red cue was automatedly extended to the entire call duration and reward time. Catch trials were not rewarded. The four monkeys were successfully trained to perform the go/no-go detection task with a mean training time of 9 ± 2.5 months. For monkeys 2 and 3, we played back audio recordings from the animal facility to maintain their motivational state during the task.

### (d) Data analysis

The first two or three consecutive recording sessions per stage per monkey were used for data analysis. In stage 1, we used the first consecutive days with at least 50 vocalizations per session. In stage 2, the first recording days after the introduction of the visual cue, and in stage 3, the first consecutive days after the introduction of the catch trial. In total, we recorded 5921 vocalizations out of which 5746 calls were used for data analysis (stage 1 = 336, stage 2 = 703, stage 3 = 4707). Call onsets were manually flagged offline using standard software (SASLab Pro version 5.2, Avisoft Bioacoustics, Germany). We classified marmoset vocalizations into previously defined groups, phee, trill, trill-phee, chirp, tsik, ekk, twitter and peep, based on their spectro-temporal profile and auditory playback [29,31]. Segmented phee calls and other rarely uttered vocalizations were excluded from the data analysis due to their intrinsic complexity [30]. The eight included call types show a very defined and distinct profile. A phee is a tone-like long call with F0 around 7–10 KHz. Phee calls are comprised of one (single phee), two (double phee) or more syllables (electronic supplementary material, figure S1*b*). Trill calls are defined by a sinusoidal-like frequency modulation (FM) throughout the call; trill-phees consist partly of a tone-like long call and partly of a sinusoidal-like FM; chirps are calls consisting of a short and descending FM sweep; a tsik is a broadband short call consisting of a linearly ascending FM sweep that merges directly into a sharply descending linear FM sweep; an ekk is a short call defined as one of the lowest frequency marmoset calls; a twitter is a short upward FM sweep; and peeps are short duration tone-like calls that have a sharply ascending or sharply descending FM. The call interval or inter-onset interval [32] represents the time between call onset to consecutive call onset and call duration represents the time between call onset to call offset. ‘1st phee’ refers to the first phee syllable and ‘2nd phee’ to the second phee syllable. For comparisons between short and long call types, we classified phees, trills and trill-phees as long affiliative calls (mean duration greater than 0.4 s according to Agamaite *et al*. [29]), while chirps, tsiks, ekks, twitters and peeps were classified as short calls (mean duration less than 0.1 s [29]). For call duration analysis, only the first syllables of phee, twitter and trill call sequences were considered.

### (e) Statistical analysis

To avoid pseudo-replication and to control for monkey-related effects, linear mixed models (LMMs) were used to assess the relationship between response variables (call interval, call duration, 1st phee duration, 2nd phee duration, long call duration, short call duration and percentage of short calls) and the fixed factor (training stage), considering monkey identity as a random effect. For call interval calculation, the LMM included call interval as a response variable, training stage as a fixed factor and monkey identity as a random effect. Robust regression was performed to reduce the effect of the outliers. To assess the overall fit between the original model and the model including the weight of the residuals, the significance of the models was tested using the Akaike information criterion and Bayesian information criterion values. The assumptions of the linear mixed model, namely linearity, normally and homoscedasticity were verified in the latter. Statistical analyses were performed using Matlab (MathWorks, Natick, MA, USA). In all performed tests, significance was tested at an *α* level of 0.05.

## 3. Results and discussion

**Figure 1.**
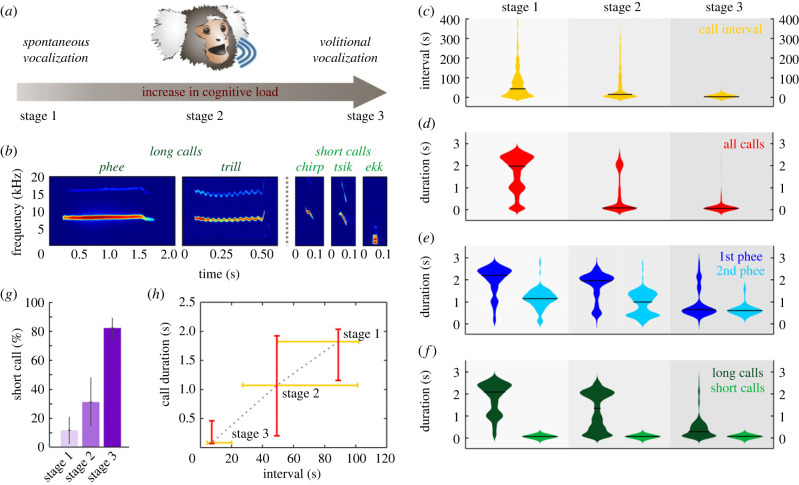
Zipf's law of brevity in vocalizing marmoset monkeys. (*a*) Schematic of the vocal conditioning paradigm with increasing cognitive load from stage 1 to stage 3. (*b*) Spectrograms of representative long (phee and trill) and short (chirp, tsik and ekk) vocalizations. Violin plots depicting the distribution of (*c*) call interval, (*d*) call duration, (*e*) 1st and 2nd phee syllable duration, and (*f*) long (phee, trill and trill-phee) and short (chirp, tsik, ekk, twitter and peep) call durations for all monkeys in stages 1, 2 and 3. Black horizontal bars indicate the medians. (*g*) Percentage of short calls across stages. (*h*) Call duration as a function of call interval across stages. Lines indicate the range from the first to the third quartiles and cross-sections indicate the medians of call duration and interval distributions across monkeys.

To analyse coding efficiency, we trained four marmoset monkeys in a vocal conditioning paradigm of increasing complexity and cognitive demand in three stages (figure 1*a*; electronic supplementary material, figure S1*a*). Animals were always rewarded regardless of call type they emitted during all training stages. Under these experimental conditions, marmoset monkeys produced several call types from their vocal repertoire, which can generally be subdivided into long call types, such as phees (single and double; electronic supplementary material, figure S1*b*) and trills, and short vocalizations, such as chirps, tsiks and ekks [29] (figure 1*b*; electronic supplementary material, figure S2). These vocalizations are mostly context specific and are produced under certain environmental conditions [29]. Phee calls are associated with long-distance communication. Trills are generally produced during foraging. Chirps and tsiks are linked to affiliative and aversive contexts, respectively, and might be uttered when the monkeys encounter their favourite food or when they detect a predator nearby. We hypothetized that long-distance contact calls, such as phee calls, would most probably be emitted during vocal training initially, when the animals separated from the rest of the colony try to reach their conspecifics. Due to their long duration, these calls maximize the likelihood of being detected; however, they are costly to produce [33]. By contrast, the use of brief vocal signals would minimize energetic costs [33]. For this, we first investigated whether call rate and duration varied through the three experimental stages to decipher potential vocal coding efficiency that developed over time despite the increasing difficulty of the task. We observed that, when progressing through training states, the monkeys significantly increased their call rate, as indicated by the decrease in call interval (estimate ± s.e.m.: −40.933 ± 0.355, *t*: −115.260, *p* < 0.0001, LMM; figure 1*c*; electronic supplementary material, figure S1*c*).

Furthermore, call duration was negatively associated with training stage. We identified a significant drop in call duration from stage 1 to stage 3 (estimate ± s.e.m.: −0.722 ± 0.002, *t*: −338.400, *p* < 0.0001, LMM; figure 1*d*; electronic supplementary material, figure S1*d*). To decipher whether the present effect was due to a decrease in the duration of distinct call types and/or exclusively to an increase in the proportion of short calls, we analysed the duration distribution of the most prominent call type in stage 1, the phee call (figure 1*e*; electronic supplementary material, figure S1*e*). To investigate changes between stages, we examined the duration distribution of single phees and first syllables of double phees (1st phee) as well as second syllables of double phees (2nd phee). We found that longer unimodal duration distributions of the phee syllables during stage 1 evolved to bimodal duration distributions with two peaks during stage 2, and finally to shorter unimodal duration distributions during stage 3 (figure 1*e*; electronic supplementary material, figure S1*e*). LMMs indicated that 1st and 2nd phee syllable durations were negatively associated with training stage (1st phee, estimate ± s.e.m.: −0.388 ± 0.118, *t*: −3.281, *p* < 0.01; 2nd phee, estimate ± s.e.m.: −0.156 ± 0.054, *t*: −2.880, *p* < 0.01, LMM). This effect was even stronger when adding other long calls such as trills and trill-phees to the data set (figure 1*f*). These results support vocal compression strategies and indicate that the proportion of long calls with shorter durations increased through the different stages of training (estimate ± s.e.m.: −0.534 ± 0.105, *t*: −5.101, *p* < 0.0001, LMM); while the duration of short calls (chirps, tsiks, ekks, twitters and peeps) remained constant (estimate ± s.e.m.: 0.003 ± 0.001, *t*: 3.641, *p* = 0.001, LMM). Finally, we analysed whether the proportion of short calls increased through the different stages of training. We found a significant increase in the occurrence of short call types from stage 1 to 3 (estimate ± s.e.m.: 35.648 ± 6.192, *t*: 5.757, *p* < 0.001, LMM; figure 1*g*; electronic supplementary material, figures S1*f* and S2), which suggests a further minimization of energetic cost with increasing vocal cognitive control. Recently, we demonstrated that marmoset monkeys are able to decouple their vocal utterances from the underlying motivational state to call on command in a vocal conditioning paradigm [20]. They were capable of initiating calls as a response to visual arbitrary cues, independently of their internal states of arousal. In this study, the experimental design may favour an increase in the number of vocalizations because calls were rewarded; despite the increase in task complexity. We observed a transition from spontaneously produced long calls with a low repetition rate in stage 1, to the production of short calls with short call intervals despite the higher cognitive demand in stage 3, reducing the energetic costs and maximizing coding efficacy (figure 1*h*; electronic supplementary material, figure S2).

Here, stages 1, 2 and 3 represent only a static image of different time points through very extensive training. Vocal transition is rather progressive and the shift towards more efficient vocal coding occurs in discrete stages between individuals. For example, call interval (electronic supplementary material, figure S1*c*) and call duration (electronic supplementary material, figure S1*d*) have a sudden drop between stages 1 and 2 for monkey 1, but takes place between stages 2 and 3 for monkeys 2 and 3. Similarly, the proportion of short calls (electronic supplementary material, figure S1*f*) increases between stage 1 and 2 for monkey 1, and between stage 2 and 3 for monkeys 2 and 3. Nevertheless, comparable compression strategies are used by all animals following the same trend. A better ability to volitionally initiate their calls on command and the monkeys' perception that calls are equally rewarded when economizing time and energy might be responsible for this variation.

Note that context-specific calls produced in stage 1, e.g. long-distance calls such as phees, are still present in stage 3 but are lower in number and with shorter duration. Additionally, both affiliative (chirps, peeps) as well as aversive (tsiks, ekks) vocalizations are produced at stage 3. If calls produced by the animals in stage 3 would still be context specific, it would have resulted in a more stereotyped calling behaviour. Instead, we identified a divergence in vocal signalling, going from a homogenous repertoire dominated by context-specific phee calls in stage 1 to a heterogeneous selection of vocalizations in stage 3 (electronic supplementary material, figure S2). Therefore, a change in calling behaviour in response to habituation to the experimental set-up is highly unlikely. Interestingly, despite the inter-individual call type variability at this last stage, calls were subjected to similar compression strategies. We propose that positive reinforcement to vocalize on command may enhance the production of more rapid and concise calls that convey accurate information in a goal-directed manner.

The gradual increase in task complexity and the high performance achieved at the end of the training [20] indicate that marmosets can take volitional control over their vocalizations. In the present study, we show that in the process to volitionally vocalize, they choose to call more frequently to obtain more reward. In addition, the animals change their vocal behaviour to emit a higher proportion of short calls and produce long vocalizations with shorter durations to save time and energy. Because of their long duration, long-distance contact calls maximize the efficacy of communication, but there is a trade-off in the energetic demands associated with producing extended vocalizations [33–35]. For example, there are biomechanical constraints on lung capacity and airflow control [36,37] and respiratory limitations [38–40], as well as higher muscular effort and significant motor control of the larynx causing higher metabolic cost [27]. Therefore, there might be a conflict between signal compression and transmission success. If producing calls of longer duration is particularly costly under the current paradigm, monkeys might attempt to produce shorter calls to economize time spent and energy consumption.

Here, it is important to point out that the calls were uttered independently of the natural triggers with which they are associated during vocal communication. We believe that the main factor that enabled these vocal compression strategies was the time spent in training, in parallel with the increase in the ability to vocalize on command. This was achieved despite the increased cognitive demands of the task through training stages. Since the marmosets displayed vocal compression at three levels, even at the more cognitively demanding stages 2 and 3, we assume that they would have developed similar strategies if they had remained at stage 1 throughout training. Performing the task in stage 2 and 3 only provides the confidence to assume that they can take volitional control over their vocal output.

Overall, our findings indicate that in accordance with Zipf's law of brevity, vocalizations that are produced more often tend to be shorter (in stage 3, approx. 82% of the vocalizations are short, figure 1*g*). When vocalizing on command, marmoset vocal signalling follows the law of least effort at three levels: (i) vocalizations are produced more frequently, (ii) longer calls exhibit shorter durations and (iii) the proportion of short calls increases. The inverse relationship between frequency of use and signal duration is explained by the principle of least effort, a rule of coding efficiency that all human languages follow [3,13,41]. The classical Chomskyan theory on language evolution assumes an anthropocentric origin of linguistic forms in the human mind and attributes an innate universal grammar to all human languages [42]. Nevertheless, the roots of our unique ability to communicate with language may have originated from the intermediate non-linguistic vocal communication systems of our primate ancestors [17]. While some studies find evidence for the law of brevity in primate vocalizations [10–12], others do not [13–15]. The field-recorded vocal repertoire of Formosan macaques showed a negative relationship between call duration and rate at different developmental stages [10]. Nevertheless, the environmental context in which calls were produced could be directly related to a particular call type and duration, and the identity of the caller was not determined. Another study on male gibbons in the wild revealed that the most commonly used notes during long-distance communication are the shortest [11]. Further, recordings from two gibbon species showed that the seven and eight different notes used by each group, respectively, support Zipf's law of brevity. However, when the most frequent note type ‘aa’ was excluded, the remaining notes did not adhere to Zipf's law. In addition, an independent study of male gibbons' solo bouts found no support for Zipf's law of abbreviation [15]. Ambiguous results have also been observed in the gestural communication of chimpanzees, where only a subset of gestures (15 out of 58) follows Zipf's law of brevity and not the entire repertoire. Similarly, Zipf's law was not found in the analyses of the entire vocal repertoire of common marmosets and golden-backed uakaris [14] but it was found in a subset of it [13]. In conclusion, while field studies yielded mixed results on the presence of Zipf's law in non-human primates, our findings clearly suggest that such vocal compression strategies may arise under selective pressure.

Studying linguistic laws in non-human primates could expand our understanding of the evolutionary origin and sequential organization of human speech in the primate lineage. Our findings offer critical insights into the humble beginnings of human language by adding a cognitive component to vocal compression and providing clues about the prevalence of linguistic laws in non-human primates. The increase in cognitive control developed in the vocal conditioning paradigm might favour the emergence of vocal coding efficiency contributing to elucidate the fundamental principles of vocal behaviour.

## Data Availability

The raw datasets and code supporting the current study have been deposited in a public repository: https://doi.org/10.5061/dryad.kprr4xh8b [43]. Supplementary material is available online [44].
